# Comparing treatment algorithms in adult traumatic brachial plexus injury

**DOI:** 10.1177/17531934261448040

**Published:** 2026-05-16

**Authors:** Justus L Groen, Ranjit D Singh, Thomas A van Essen, Martijn JA Malessy, Willem Pondaag

**Affiliations:** 1 Department of Neurosurgery, Leiden Nerve Center, Leiden University Medical Center, Leiden, the Netherland; 2 University Neurosurgical Center Holland, Leiden University Medical Center, Haaglanden Medical Center and HAGA Teaching Hospital, Leiden and The Hague, the Netherlands; 3 Leiden University Medical Center, Leiden, the Netherlands

Dear Editor,

We would like to bring to your attention a collaborative research initiative ‘Comparing Treatment Algorithms in Adult Traumatic Brachial Plexus Injury’ (CTA-BPI). This multicentre study addresses a significant knowledge gap in the current management of adult traumatic brachial plexus injury (ATBPI), where treatment algorithms vary widely (Shin et al., 2022; Weekes et al., 2025) and high-quality comparative evidence is lacking. Currently, no studies have comprehensively evaluated functional and quality of life outcomes across different treatment approaches in specialist centres.

Adult patients with a severe complete or incomplete brachial plexus injury after high-impact trauma are eligible for this study. Treatment strategies for ATBPI may involve a combination of nerve grafting, nerve transfers, tendon transfers and free muscle transfers, performed at various intervals after trauma. The aim is to compare treatment algorithms for restoration of arm and hand function, including early vs. delayed surgical intervention (Pondaag et al., 2019). We plan a prospective observational comparative-effectiveness study with a target trial emulation framework (Cashin et al., 2025). We will start with profiling each participating centre’s real-world treatment algorithm and case-mix, using a predefined number of treatment strategies, including early nerve repair-dominant algorithms, delayed nerve transfers, secondary reconstruction-dominant algorithms and muscle-transfer-focused algorithms. Outcomes will be compared between strategies using hierarchical and causal-inference methods while accounting for clustering and confounding.

This prospective, observational international multicentre study will leverage the variety of treatment preferences across specialized nerve and reconstructive surgical centres worldwide (Shin et al., 2022). After appropriate protocol development and centre identification, patient recruitment is planned to begin in October 2026.

To ensure that variation in practice becomes the main exposure, inclusion criteria and outcome measures will be standardized between centres. Outcomes will include the Brachial Assessment Tool, MRC grading, range of motion, Brief Pain Inventory, EQ-5D assessing health-related quality of life and safety assessments, to allow appropriate comparison in keeping with the consensus statements on outcome measurement in ATBPT (Miller et al., 2024; Wilson et al., 2024). As recovery time after ATBPI surgery is long, we plan to measure outcomes regularly up to 36 months after surgery.

We believe that this research initiative could significantly impact clinical practice by establishing evidence-based treatment strategies, ultimately improving functional and quality of life outcomes for patients with ATBPI.

We welcome collaboration from recognized nerve and reconstructive surgical centres treating ATBPI. We encourage interested nerve surgeons to visit our website and sign up (https://www.leidennervecenter.org/compare-treatment-atbi) or email info@leidennervecenter.org.

Sincerely,
